# Mental and Behavioral Disorders Due to Substance Abuse and Perinatal Outcomes: A Study Based on Linked Population Data in New South Wales, Australia

**DOI:** 10.3390/ijerph110504991

**Published:** 2014-05-08

**Authors:** Michelle R. Bonello, Fenglian Xu, Zhuoyang Li, Lucy Burns, Marie-Paule Austin, Elizabeth A. Sullivan

**Affiliations:** 1 Unit of National Perinatal Epidemiological and Statistics, School of Women’s and Children’s Health, University of New South Wales, Sydney 2031, Australia; E-Mails: zhuoyang.li@unsw.edu.au (Z.L.); e.sullivan@unsw.edu.au (E.A.S); 2 National Drug and Alcohol Research Centre (NDARC), University of New South Wales, Sydney 2031, Australia; E-Mails: f.xu@unsw.edu.au (F.X.); l.burns@unsw.edu.au (L.B.); 3 Perinatal and Women’s Mental Health Research Unit, St. John of God Health Care and School of Psychiatry, University of New South Wales, Sydney 2052, Australia; E-Mail: m.austin@unsw.edu.au (M.-P.A.)

**Keywords:** pregnancy, perinatal outcomes, substance use, alcohol, opioids, cannabinoids

## Abstract

*Background:* The effects of mental and behavioral disorders (MBD) due to substance use during peri-conception and pregnancy on perinatal outcomes are unclear. The adverse perinatal outcomes of primiparous mothers admitted to hospital with MBD due to substance use before and/or during pregnancy were investigated. *Method:* This study linked birth and hospital records in NSW, Australia. Subjects included primiparous mothers admitted to hospital for MBD due to use of alcohol, opioids or cannabinoids during peri-conception and pregnancy. *Results:* There were 304 primiparous mothers admitted to hospital for MBD due to alcohol use (MBDA), 306 for MBD due to opioids use (MBDO) and 497 for MBD due to cannabinoids (MBDC) between the 12 months peri-conception and the end of pregnancy. Primiparous mothers admitted to hospital for MBDA during pregnancy or during both peri-conception and pregnancy were significantly more likely to give birth to a baby of low birthweight (AOR = 4.03, 95%CI: 1.97–8.24 for pregnancy; AOR = 9.21, 95%CI: 3.76–22.57 both periods); preterm birth (AOR = 3.26, 95% CI: 1.52–6.97 for pregnancy; AOR = 4.06, 95%CI: 1.50–11.01 both periods) and admission to SCN or NICU (AOR = 2.42, 95%CI: 1.31–4.49 for pregnancy; AOR = 4.03, 95%CI: 1.72–9.44 both periods). Primiparous mothers admitted to hospital for MBDO, MBDC or a combined diagnosis were almost three times as likely to give birth to preterm babies compared to mothers without hospital admissions for psychiatric or substance use disorders. Babies whose mothers were admitted to hospital with MBDO before and/or during pregnancy were six times more likely to be admitted to SCN or NICU (AOR = 6.29, 95%CI: 4.62–8.57). *Conclusion:* Consumption of alcohol, opioids or cannabinoids during peri-conception or pregnancy significantly increased the risk of adverse perinatal outcomes.

## 1. Introduction

The prevalence of substance use has increased markedly in the past two decades and women are not immune to this trend, with approximately 90% of drug abusing women being of reproductive age and increasingly including pregnant women [1]. Alcohol and substance use has been found to result in adverse perinatal outcomes including preterm birth and low birthweight which have been subsequently associated with long term infant morbidity and mortality [2,3,4]. Alcohol use during pregnancy has been associated with preterm birth, low APGAR scores, admission to special care nurseries (SCN) and fetal alcohol spectrum disorders [5,6,7,8]. 

The literature suggests that opiate use is associated with a range of adverse clinical outcomes including preterm birth, poor growth, and increased length of hospital stay for the baby [5,9,10,11]. Neonatal outcomes from cannabis exposure has been less conclusive with a number of studies reporting adverse effects including low birthweight [12] and preterm birth [12] and other studies dispute these findings [13]. A population-based self-report study in the United States found cannabis use was not associated with low birthweight or preterm birth [13] whereas a self-report study in Australia found that cannabis use was associated with low birthweight, preterm labour and admission to neonatal intensive care unit (NICU) [12]. A recent overview of studies on prenatal cannabis exposure and infant outcomes has concluded that fetal development is affected by prenatal maternal cannabis use [14]. Polysubstance use is common among pregnant women and the effects on perinatal outcomes are more significant. A study in Denmark reported that polysubstance use was associated with poorer perinatal outcomes compared with only alcohol or with no substance exposure [15]. 

The pregnancy period represents a time of significant change and adjustment in women’s lives [16]. However the significance of substance use during peri-conception and the risk of adverse neonatal outcomes are largely unknown. Substance use is often a chronic disorder where relapse is common. Therefore the peri-conception period may be relevant clinically to women of childbearing age. An admission for a mental and behavioral disorder (MBD) due to substance use in a women of childbearing age may present an opportunity to identify women at risk of substance use during pregnancy and provide an opportunity to discuss family planning and available resources for substance use treatment with the view of minimising or preventing substance use and/or unplanned pregnancy. 

The majority of studies to date on substance use involve self-reported information of which the limitations are well documented and include under-reporting and recall bias [17]. The dispute related to the validity of self-reported data is no greater than in alcohol and drug research [18]. Hospital admission records and other administrative data provide an objective measure of severe substance use in pregnancy and complements self-reported data and other similar methodologies. Population-based administrative data provide a mechanism for validation of self-reported observations and trend data over time.

This study investigates women who were admitted to hospital with a primary diagnosis MBD due to substance use in the period 12 months before and/or during pregnancy. The aims of the study are to measure the impact of MBD due to substance use (alcohol, cannabinoids and opioids) necessitating hospital admission before and during pregnancy on perinatal outcomes and to analyse the impact of poly-substance use of MBD due to alcohol, opioids and cannabinoids on pregnancy outcomes.

## 2. Methods

### 2.1. Study Design

This is a retrospective cohort study using population linked data. Baby birth records of primiparous women from 1 January 2003 to 31 December 2006 (from the New South Wales (NSW) Midwives Data Collection (MDC)) were linked with maternal hospital records (from the NSW Admitted Patients Data Collection (APDC)). About ten per cent (13,113 mothers) of women who gave birth (MDC records) and did not have a diagnosis of psychiatric illness and substance use were randomly selected as the comparison group.

The MDC is a population-based data collection of all births in New South Wales, Australia. It covers all births of at least 20 weeks gestation or at least 400g birth weight in public and private hospitals as well as home births, and includes information on maternal characteristics, pregnancy, labour, delivery and perinatal outcomes. The APDC is a routinely collected census of all hospital separations. It includes all patient hospitalizations from NSW public and private hospitals (including psychiatric hospitals) and day procedures. Information on patients includes demographics, diagnoses and clinical procedures. Since 1999, diagnoses have been coded according to the 10th revision of the International Statistical Classification of Diseases and Related Health Problems, Australian Modification (ICD-10-AM) [19]. Data linkage was performed by the Centre for Health Record Linkage (CHeReL) using probabilistic record linkage methods and Choicemaker software [20]. Linkage quality was assessed by a review from a random sample of 1,000 records. The false positive rate of the linkage was 0.3% and false negative less than 0.5%. 

### 2.2. Study Population

The study subjects (945) included all primiparous women who gave birth in NSW between 1 January 2003 to 31 December 2006 and were admitted to hospital with a diagnosis of mental and behavioral disorders due to use of alcohol, opioids and cannabinoids in the period from the 12th month before pregnancy to the end of pregnancy. The comparison group included a 10% sample of primiparous mothers with no documented diagnosis of psychiatric illness or substance use during the study period. All mothers were aged between 18 and 44 years old.

### 2.3. Definition

The diagnosis of mental and behavioral disorders due to use of alcohol include principal, stay and other diagnoses of ICD-10-AM F10.

The diagnosis of mental and behavioral disorders due to use of opioids include principal, stay and other diagnoses of ICD-10-AM F11.

The diagnosis of mental and behavioral disorders due to use of cannabinoids include principal, stay and other diagnoses of ICD-10-AM F12.

Low birthweight: birthweight of less than 2,500 grams (<2,500 grams).

Preterm birth: birth of less than 37 weeks of gestation (<37 weeks).

### 2.4. Statistical Analysis

Logistic regression was used to measure the risk of adverse perinatal outcomes due to use of alcohol, opioids and cannabinoids for low birthweight, preterm birth and admission to SCN or NICU. Odds ratios were adjusted for maternal age, maternal country of birth, smoking status (yes/no), remoteness of living area, and a socioeconomic indicator (the Index of Relative Socio-economic Disadvantage) [21], pre-existing maternal diabetes, pre-existing maternal hypertension, pregnancy complications (pre-eclampsia, gestational diabetes), method of birth, infant gender and fetal/neonatal death. All analyses were conducted using IBM SPSS Statistics 20.

### 2.5. Ethics Approval

This research was approved by the NSW Population & Health Services Research Ethics Committee and the Human Research Ethics Committee of the University of New South Wales, Sydney, Australia.

## 3. Results

From 1 January 2003 to 31 December 2006 there were 945 primiparous mothers admitted to hospital for a MBD due to alcohol, opioid or cannabinoid use between 12 months peri-conception period and the end of pregnancy in NSW. Among them, 304 primiparous mothers admitted to hospital for MBD due to alcohol use (MBDA), 306 primiparous mothers for MBD due to opioids use (MBDO) and 497 primiparous mothers for MBD due to cannabinoids (MBDC). There were 146 admissions were diagnoses overlapped (that it, one admission included more than one diagnosis). For example, there were 16 admissions for the combined diagnosis of alcohol and opioids; 52 admissions for the combined diagnoses of alcohol and cannabinoids; 62 admissions for the combined diagnoses of opioids and cannabinoids and 16 admissions for the combined diagnoses of alcohol, opioids and cannabinoids. The comparison group included 13,113 primiparous mothers (10%) who were not admitted to hospital with a diagnosis of psychiatric or substance use disorder during the study period. There were 20 birth records with birthweight values missing (12 in comparison group and eight in hospital admission group) and one birth in the comparison group with a missing value for gestational age.

### 3.1. Hospital Admission for Mental and Behavioral Disorders due to Substance Use and Perinatal Outcomes

Logistic regression model was used for 220 mothers who were admitted to hospital with MBDA, 212 mothers with MBDO, 367 mothers with MBDC, 146 mothers with MBD due to poly-substance use which refers to more than one use of alcohol, opioids and cannabinoids in the period 12 months before pregnancy and during pregnancy. 

The diagnoses of MBDA, MDBO, MBDC and poly-substance use before and/or during pregnancy on perinatal outcomes including low birthweight, preterm birth and baby admission to an NICU or SCN are presented in Table 1. 

Compared with mothers who were not admitted to hospital with a diagnosis of a psychiatric disorder or substance use, mothers admitted to hospital with an alcohol-related diagnosis of MBDA were almost twice as likely to give birth of babies with low birthweight (adjusted odds ratio AOR = 1.97, 95%CI: 1.26–3.06); mothers with MBDO and MBDC were about four times more likely to give birth to low birthweight babies (AOR for MBDO and MBDC were 3.72 (95%CI: 2.57–5.39), 4.31 (95%CI: 3.20–5.82) respectively). Mothers with poly-substance use of more than one of MBDA, MBDO or MBDC were three times as likely to give birth to a low birthweight baby (AOR = 3.21, 95%CI: 2.03–5.08).

Mothers admitted to hospital with a diagnosis of MBDO, MBDC or combined diagnosis were almost three times as likely to give birth to preterm babies compared to mothers without hospital admissions for psychiatric or substance use disorders. 

Babies whose mothers were admitted to hospital with MBDO before and/or during pregnancy were six times as likely to be admitted to SCN or NICU (AOR = 6.29, 95%CI: 4.62–8.57). Babies whose mothers where admitted to hospital with MBDC or a poly-substance use diagnosis was two and four times more likely to be admitted to SCN or NICU respectively. 

**Table 1:** Mental disorder due to alcohol, opioids or cannabinoids and perinatal outcomes.

Perinatal Outcomes	Mental and Behavioral Disorders due to Substance Use	n	OR	95%CI		AOR ^a^	95%CI	
Low birthweight (<2,500 grams)	No substance	13,101	1			1		
	Alcohol *	219	2.01	1.32	3.04	1.97	1.26	3.06
	Opioids *	212	4.97	3.61	6.83	3.72	2.57	5.39
	Cannabinoids *	367	5.43	4.26	6.91	4.31	3.20	5.82
	Polysubstance ^b,^*	146	4.02	2.68	6.01	3.21	2.03	5.08
Preterm birth (<37 weeks)	No substance	13,112	1			1		
	Alcohol	220	1.28	0.79	2.05	1.29	0.78	2.15
	Opioids *	212	3.34	2.36	4.71	2.99	2.00	4.46
	Cannabinoids *	367	2.91	2.20	3.83	2.68	1.92	3.74
	Polysubstance ^b,^*	146	3.35	2.22	5.05	2.97	1.84	4.78
Admission to SCN or NICU	No substance	13,113	1			1		
	Alcohol	220	1.13	0.82	1.57	1.10	0.77	1.57
	Opioids *	212	6.66	5.04	8.81	6.29	4.62	8.57
	Cannabinoids *	367	2.35	1.89	2.92	2.15	1.67	2.76
	Polysubstance ^b,^*	146	4.29	3.09	5.95	4.03	2.80	5.78

Notes: OR: crude odds ratio. AOR: adjusted odds ratio. SCN: special care nursery; NICU: neonatal intensive care unit. **^a^**: Adjusted for maternal age, pre-existing maternal diabetes, pre-existing maternal hypertension, pregnancy complications (pre-eclampsia, gestational diabetes), maternal smoking status (yes/no), remoteness of living area, Index of Relative Socio-economic Disadvantage, maternal country of birth, delivery method, infant gender and fetal/neonatal death. **^b^**: Poly-substance refers to overlapped diagnoses among MBDA, MBDO and MBDC. At least two of these disorders were diagnosed in the 12-month period before pregnancy and during pregnancy. * Significantly associated with perinatal outcomes compared with non-substance use (*p* < 0.05).

### 3.2. Hospital Admission Time for MBD and Low Birthweight

The impact of a diagnosis of MBD due to substance use before and during pregnancy and the birthweight outcomes are presented in Table 2. Mothers admitted to hospital with MBDA during pregnancy or in both peri-conception and pregnancy periods were significantly more likely to give birth to a baby of low birthweight (AOR = 4.03, 95%CI: 1.97–8.24 for pregnancy; AOR = 9.21, 95%CI: 3.76–22.57 for both periods). Admission for MBDA in peri-conception was found not to be significantly associated with low birthweight babies.

**Table 2:** Mental and behavioral disorders due to substance use in peri-conception and pregnancy and low birthweight.

Mental and Behavioral Disorders due to Substance Use	Period of Substance Use	n	OR	95%CI		AOR ^a^		95%CI			
				Lower	Upper			Lower		Upper	
Alcohol use	No	13,101	1			1					
	Peri-conception	226	1.61	1.03	2.51	1.53		0.95		2.45	
	Pregnancy *	54	4.26	2.23	8.12	4.03		1.97		8.24	
	Both ^**b**,^*	23	11.46	5.01	26.22	9.21		3.76		22.57	
Opioids use	No	13,101	1			1					
	Peri-conception *	62	3.21	1.67	6.19	2.27		1.10		4.71	
	Pregnancy *	185	5.07	3.62	7.12	3.56		2.39		5.32	
	Both *	59	7.08	4.08	12.27	5.71		3.15		10.35	
Cannabinoids use	No	13,101	1			1				
	Peri-conception *	140	3.24	2.09	5.02	2.72	1.67		4.44	
	Pregnancy *	330	6.02	4.70	7.72	4.85	3.55		6.62	
	Both *	27	3.39	1.28	8.96	3.13	1.11		8.82	

Notes: OR: crude odds ratio. AOR: adjusted odds ratio. SCN: special care nursery; NICU: neonatal intensive care unit. **^a^**: Adjusted for maternal age, pre-existing maternal diabetes, pre-existing maternal hypertension, pregnancy complications (pre-eclampsia, gestational diabetes), maternal smoking status (yes/no), remoteness of living area, Index of Relative Socio-economic Disadvantage, maternal country of birth, delivery method, infant gender and fetal/neonatal death. **^b^**: Both includes the periods of 12 months before pregnancy and pregnancy. * Significantly associated with low birthweight compared with non-substance use (*p* < 0.05).

Mothers admitted to hospital with MBDO before or during pregnancy, or in both periods, were at significantly increased risk of giving birth to a baby with low birthweight (AOR = 2.27, 95%CI: 1.10–4.71 for peri-conception; AOR = 3.56, 95%CI: 2.39–5.32 for pregnancy and AOR = 5.71, 95%CI: 3.15–10.35 for both periods). Similarly, mothers admitted to hospital with a diagnosis of MBDC before or during pregnancy, or in both periods were more likely to give birth to a low birthweight baby (AOR = 2.72, 95%CI: 1.67–4.44 for peri-conception; AOR = 4.85, 95%CI: 3.55–6.62 for pregnancy and AOR = 3.13, 95%CI: 1.11–8.82 for both periods). 

### 3.3. Hospital Admission Time for MBD and Preterm Birth

The impact of a diagnosis of MBD due to substance use before and during pregnancy and the preterm birth outcomes are presented in Table 3. Compared with mothers who were not admitted to hospital with a diagnosis of psychiatric disorder or substance use, mothers admitted to hospital with MBDA during pregnancy or in both periods were more likely to give birth to a preterm baby (AOR = 3.26, 95%CI: 1.52–6.97 for pregnancy; AOR = 4.06, 95%CI: 1.50–11.01 for both periods). Admission for MBDA in peri-conception was found not to be significantly associated with preterm births.

The risk of a preterm birth increased significantly when mothers were admitted to hospital for MBDO during pregnancy or in both periods of peri-conception and pregnancy (AOR = 3.22, 95%CI: 2.11–4.92 for peri-conception; AOR = 5.15, 95%CI: 2.76–9.63 for both periods). Mothers admitted to hospital with MBDC during pregnancy were more likely to give birth to preterm babies (AOR = 3.61, 95%CI: 2.58–5.04). Peri-conception admission for MBDO and MBDC were not found to be significantly associated with preterm birth however admission for MBDC during pregnancy was significantly associated (AOR = 3.61, 95%CI: 2.58–6.04).

**Table 3:** Mental and behavioral disorders due to substance use in peri-conception and pregnancy and preterm birth.

Mental and Behavioral Disorders due to Substance Use	Period of Substance Use	n	OR	95%CI		AOR ^a^	95%CI	
				Low	Up		Low	Up
Alcohol use	No	13,112	1					
	Peri-conception	226	1.10	0.67	1.81	1.07	0.62	1.83
	Pregnancy *	55	3.38	1.74	6.56	3.26	1.52	6.97
	Both ^b,^*	23	4.77	1.87	12.12	4.06	1.50	11.01
Opioids use	No	13,112						
	Peri-conception	62	1.72	0.78	3.78	1.38	0.56	3.37
	Pregnancy *	185	3.73	2.61	5.32	3.22	2.11	4.92
	Both *	59	5.02	2.82	8.96	5.15	2.76	9.63
Cannabinoids use	No	13,112						
	Peri-conception	140	1.62	0.94	2.78	1.56	0.87	2.80
	Pregnancy *	330	3.77	2.88	4.94	3.61	2.58	5.04
	Both	27	2.35	0.81	6.81	1.92	0.55	6.76

Notes: OR: crude odds ratio. AOR: adjusted odds ratio. **^a^**: Adjusted for maternal age, pre-existing maternal diabetes, pre-existing maternal hypertension, pregnancy complications (pre-eclampsia, gestational diabetes), maternal smoking status (yes/no), remoteness of living area, Index of Relative Socio-economic Disadvantage, maternal country of birth, delivery method, infant gender and fetal/neonatal death. **^b^**: Both includes the periods of 12 months before pregnancy and pregnancy. * Significantly associated with low birthweight compared with non-substance use (*p* < 0.05).

### 3.4. Hospital Admission Time for MBD and Babies’ Admission to SCN or NICU

The impact of a diagnosis of MBD due to substance use before and during pregnancy and babies’ admission to SCN or NICU outcomes are presented in Table 4. Babies were more likely to be admitted to SCN or NICU if their mothers were admitted to hospital with MBDA during pregnancy or in both peri-conception and pregnancy (AOR = 2.42, 95%CI: 1.31–4.49 for pregnancy; AOR = 4.03, 95%CI: 1.72–9.44 for both periods). Admission for MBDA in peri-conception was found not to be significantly associated with baby’s admission to SCN or NICU.

The risk of admission to SCN or NICU increased significantly if the mother was admitted to hospital for MBDO before pregnancy, during pregnancy or in both periods (AOR = 2.53, 95%CI: 1.48–4.32 for peri-conception; AOR = 6.89, 95%CI: 4.93–9.63 for pregnancy; AOR = 12.94, 95%CI: 6.94–24.14 for both periods). Similarly, babies were more likely to be admitted to SCN or NICU if their mothers were admitted to hospital with MBDC before pregnancy, during pregnancy or in both periods (AOR = 1.99, 95%CI: 1.36–2.92 peri-conception; AOR = 2.84, 95%CI: 2.19–3.68 for pregnancy; AOR = 4.83, 95%CI: 2.11–11.05 for both periods). 

**Table 4:** Mental and behavioral disorders due to substance use in peri-conception and pregnancy and baby’s admission to SCN or NICU.

Mental and Behavioral Disorders due to Substance Use	Period of Substance Use	n	OR	95%CI		AOR ^a^	95%CI	
				Low	Up		Low	Up
Alcohol use	No	13,113	1			1		
	Peri-conception	226	1.16	0.84	1.59	1.03	0.72	1.47
	Pregnancy *	55	2.45	1.41	4.25	2.42	1.31	4.49
	Both ^b,^*	23	4.67	2.06	10.61	4.03	1.72	9.44
Opioids use	No	13,113	1			1		
	Peri-conception *	62	2.71	1.62	4.52	2.53	1.48	4.32
	Pregnancy *	185	7.37	5.45	9.97	6.89	4.93	9.63
	Both *	59	12.57	6.98	22.62	12.94	6.94	24.14
Cannabinoids use	No	13,113	1			1		
	Peri-conception *	140	2.17	1.52	3.08	1.99	1.36	2.92
	Pregnancy *	330	2.89	2.31	3.62	2.84	2.19	3.68
	Both *	27	6.23	2.89	13.45	4.83	2.11	11.05

Notes: OR: crude odds ratio. AOR: adjusted odds ratio. SCN: special care nursery; NICU: neonatal intensive care unit. ^a^: Adjusted for maternal age, pre-existing maternal diabetes, pre-existing maternal hypertension, pregnancy complications (pre-eclampsia, gestational diabetes), maternal smoking status (yes/no), remoteness of living area, Index of Relative Socio-economic Disadvantage, maternal country of birth, delivery method, infant gender and fetal/neonatal death. ^b^: Both includes the periods of 12 months before pregnancy and pregnancy. *Significantly associated with low birthweight compared with non-substance use (*p* < 0.05).

The mother’s demographic factors and characteristics for case and comparison groups is detailed in Table 5.

**Table 5:** Characteristics of mothers with and without hospital admissions for substance use.

Variables	Values	n	Substance Use Group		Control Group		*p* value
			Mother	%	Mother	%	
Smoking during pregnancy	No	12,030	275	29.16	11,755	90.01	<0.01
	Yes	1,972	668	70.84	1304	9.99	
	Total	14,002	943	100	13,059	100	
Index of Relative Socio-economic Disadvantage	The least disadvantaged group	3,055	111	11.85	2,944	22.69	<0.01
	2	3,231	177	18.89	3,054	23.54	
	3	2,541	233	24.87	2,308	17.79	
	4	2,201	192	20.49	2,009	15.49	
	The most disadvantaged group	2,882	224	23.91	2,658	20.49	
	Total	13,910	937	100	12,973	100	
Country of birth	Australia	10,040	848	89.74	9,192	70.1	<0.01
	Other countries	4,018	97	10.26	3,921	29.9	
	Total	14,058	945	100	13,113	100	
Remoteness of living area	Major city	9,478	569	60.73	8,909	68.67	<0.01
	Regional area	4,316	358	38.21	3,958	30.51	
	Remote area	116	10	1.07	106	0.82	
	Total	13,910	937	100	12,973	100	
Maternal age	<20	841	178	18.84	663	5.06	<0.01
	20–24	2,848	391	41.38	2,457	18.74	
	25–29	4,385	207	21.9	4,178	31.86	
	30–34	4,165	111	11.75	4,054	30.92	
	35–39	1,548	47	4.97	1,501	11.45	
	40–45	271	11	1.16	260	1.98	
	Total	14,058	945	100	13,113	100	
Delivery method	Vaginal	9,900	705	74.6	9,195	70.15	<0.01
	Caesarean section	4,153	240	25.4	3,913	29.85	
	Total	14,053	945	100	13,108	100	
Gestational diabetes	No	13,503	930	98.41	12,573	95.88	<0.01
	Yes	555	15	1.59	540	4.12	
	Total	14,058	945	100	13,113	100	
Maternal diabetes mellitus	No	13,979	934	98.84	13,045	99.48	=0.01
	Yes	79	11	1.16	68	0.52	
	Total	14,058	945	100	13,113	100	
Maternal hypertension	No	13,898	938	99.26	12,960	98.83	>0.05
	Yes	160	7	0.74	153	1.17	
	Total	14,058	945	100	13,113	100	
Pre-eclampsia	No	11,757	846	94.00	10,911	92.54	>0.05
	Yes	934	54	6.00	880	7.46	
	Total	12,691	900	100	11,791	100	

## 4. Discussion

This study demonstrates that women who are admitted to hospital for a MBD due to substance abuse during pregnancy or in the peri-conception period, here defined as 12 months preceding pregnancy, for alcohol, opioids or cannabinoids are at significantly increased risk of giving birth to a baby with poorer perinatal outcomes compared to women who did not have a diagnosis of psychiatric illness or substance abuse. This study found more mothers were admitted to hospital with MBD due to alcohol use before the pregnancy period compared with during the pregnancy period. This suggests that some women are able to reduce or control alcohol use during pregnancy and is consistent with the literature. 

The detrimental effects of alcohol on a growing fetus are well established, extensive and can be life-long. This study found that mothers admitted to hospital with MBD due to alcohol (MBDA) during pregnancy or in both peri-conception and pregnancy were more likely to have babies there were low birthweight, preterm and be admitted to SCN or NICU. The risk increased when mothers had a hospital admission for MBDA during pregnancy, and both before and during pregnancy. In this study, alcohol use both during and before pregnancy was found to result in a synergistic effect with an increased risk of nearly 10 times on birthweight of the baby. Studies supporting this have observed that newborns exposed primarily to alcohol in utero were at a significant risk of being born low birthweight [15] and that the level of maternal drinking prior to pregnancy was found to be significantly related to preterm birth [22] suggesting alcohol consumption in the peri-conception period and during pregnancy may result in significant adverse perinatal outcomes. 

For babies whose mother was admitted to hospital with a diagnosis of MBD due to opioids (MBDO) in both peri-conception and pregnancy periods the increased risk of admission to SCN or NICU was more than the sum of the effects of either peri-conception or pregnancy. This indicates that opioid use before and during pregnancy has a synergistic effect on perinatal outcomes. Opiate use in the peri-conception and pregnancy periods significantly increased the risk of giving birth to a low birthweight baby. Long-term abstinence for opiate users is unusual and agonist treatment is advised over detoxification for pregnant women [23] which may be a contributing factor to the increased risk of adverse perinatal outcomes in this study for those admitted with MBDO compared to the rest of the cohort who may undergo detoxification.

This study found that cannabinoids use during pregnancy increased the risk of a low birthweight baby, preterm birth and admission to SCN or NICU. This finding was consistent with previous studies suggesting cannabinoids can adversely affect perinatal outcomes [12,14]. Cannabinoids are often mixed with tobacco when smoked and may confound the detrimental effects of use of this drug.

It is well established that dependent substance users are often dependent on other drugs. A study of women who were found by objective measure to have heavy alcohol use during pregnancy were also significantly more likely to use other drugs of abuse [24]. This study found poly-substance use significantly increased the risk of low birthweight, preterm birth and admission to SCN or NICU. These results are similar to a study that found women with poly-drug use were at increased risk of giving birth to a pre-term baby, low birthweight baby or baby with neonatal abstinence syndrome compared to alcohol alone [15]. A study found that pregnant women who used drugs and alcohol were less likely to cease substance use compared to pregnant women who used drugs or alcohol alone [25]. Pregnant women who receive no antenatal care may be at higher risk for substance use and poly-substance use with a study finding 52% of unbooked women positive for cocaine and 23% of these positive for poly-substance use [26] which could provide an indicator for clinicians. These results suggest that women with poly-substance use may find reducing or ceasing substance use more difficult and the effect on adverse perinatal outcomes more significant. This group of women proposes a particular challenge to healthcare providers.

The results of this study suggest that peri-conception opioids and cannabinoids significantly increased the risk of low birthweight and admission to SCN or NICU. A study based on self-reported data found that high levels of alcohol and illicit drug use in the peri-conception period were predictors for continuation during pregnancy compared to moderate use [25] suggesting that women with heavy substance use are at significant risk of substance use during pregnancy. A maternal mental health study concluded that the period before pregnancy and during pregnancy is a sensitive developmental period for both the mother and baby for future emotional, behavioural and mental development [27]. 

The results from this study indicate that the peri-conception period may be an important period and affect perinatal outcomes. These results have clinical implications that suggest substance use history should be assessed in all pregnant women and appropriate management instituted. In the first instance, the clinical implications are that women of reproductive age admitted for MBD due to substance use should be proactively offered family planning options before discharge from hospital and a follow up plan due to the markedly increased risk of substance use during pregnancy. Women of reproductive age admitted for MBD due to substance use should be counselled on the increased risk of adverse perinatal outcomes if she became pregnant.

The population in this study was women admitted to hospital for MBD due to substance abuse before or in the peri-conception period which suggests the illness was significant enough to require hospital admission. Research on this population is not extensive and therefore the effects of drug exposure before and during pregnancy are still not fully understood and further research on this population is warranted. From a public health perspective, repeating this study in five to ten years with contemporary data would provide some interesting insights into the trends of women admitted to hospital with an MBD due to substance use to determine if there is an increase in the number of admissions. This method of surveillance research can provide insights into population trends and whether public health prevention strategies should be implemented. Future research will be conducted on the characteristics of these women to determine and may provide important clinical indications for women with substance use issues who may be at increased risk of giving birth to a baby with poor neonatal outcomes. 

This study examined perinatal outcomes from mothers with a history of inpatient admission for MBD due to substance abuse and these results may not be generalised to those with MBD who are not hospitalised. These results are more likely to reflect women with relatively heavy use of substances that result in hospitalisation and it would be of value in future studies to have community based data (non-hospital admission) for comparison.

This study population is likely to be significantly different to a population who may respond to surveys or provide accurate self-reported information and comparisons with research using this methodology should take this into account. Population data provides an objective measure of substance use and complements survey and self-reported data which has limitations.

These results provide clinical and epidemiological evidence that suggest women admitted for a MBD due to substance use during peri-conception or during pregnancy are at increased risk of giving birth to baby with adverse perinatal outcomes. These results suggest that women of reproductive age admitted with an MBD due to substance use should be appropriately managed at discharge with information on the perinatal outcome risks and should be managed with substance use treatment and family planning options. This study also provides a quantitative analysis of how many women were admitted to hospital in NSW for an MBD due to substance use and the risk to perinatal outcomes. 

### Limitations

The scope of this study was limited to women admitted to hospital with mental and behavioral disorders due to psychoactive substances. There is a well-established relationship between substance use and other forms of psychiatric illness and the interplay of these conditions is complex. The individual effects of substance use or mental and behavioral disorders as well as the interplay between substance use and mental illness will be studied further in future.

A limitation of this study is the lack of information on the level of exposure of alcohol, cannabinoids and opioids in the peri-conception and pregnancy periods. In addition, women admitted to hospital with MBD due to substance use are likely to differ significantly from other women who may use alcohol and illicit substances before and during pregnancy and not be admitted to hospital with MDB, further investigation into substance use and perinatal outcomes is warranted.

## 5. Conclusions

Severe consumption of alcohol, opioids or cannabinoids before and during pregnancy significantly increased the adverse pregnancy outcomes. Attention should be extended from pregnancy to peri-conception for preventing or reducing substance use.

## References

[1] Kuczkowski K. (2003). Anesthetic implications of drug abuse in pregnancy. J. Clin. Anesth..

[2] King-Hele S., Webb R.T., Mortensen P.B., Appleby L., Pickles A., Abel K.M. (2009). Risk of stillbirth and neonatal death linked with maternal mental illness: A national cohort study. Arch. Dis. Child.-Fetal Neonatal.

[3] Tucker J., McGuire W. (2004). ABC of preterm birth Epidemiology of preterm birth. Brit. Med. J..

[4] Gyllstrom M.E., Hellerstedt W.L., McGovern P.M. (2010). Indepdendent and interactive associations of prenatal mood and substance use with infant birth outcomes. Matern. Child Health J..

[5] Burns L., Mattick R.P., Cooke M. (2006). Use of record linkage to examine alcohol use in pregnancy. Alcohol. Clin. Exp. Res..

[6] Mullally A., Cleary B.J., Barry J., Fahey T.P., Murphy D.J. (2011). Prevalence, predictors and perinatal outcomes of peri-conceptional alcohol exposure—Retrospective cohort study in an urban obstetric population in Ireland. BMC Preg. Childbirth.

[7] Ornoy A., Ergaz Z. (2010). Alcohol abuse in pregnant women: Effects on the fetus and newborn, mode of action and maternal treatment. Int. J. Environ. Res. Public Health.

[8] Patra J., Bakker R., Irving H., Jaddoe V.W.V., Malini S., Rehm J. (2011). Dose-response relationship between alcohol consumption before and during pregnancy and the risks of low birthweight, preterm birth and small for gestational age (SGA)—A systematic review and meta-analyses. BJOG.

[9] Minnes S., Lang A., Singer L. (2011). Prenatal tobacco, marijuana, stimulant, and opiate exposure: Outcomes and practice implications. Addict. Sci. Clin. Pract..

[10] Wouldes T.A., Woodward L.J. (2010). Maternal methadone dose during pregnancy and infant clinical outcome. Neurotoxicol. Teratol..

[11] Burns L., Mattick R.P., Lim K., Wallace C. (2007). Methadone in pregnancy: Treatment retention and neonatal outcomes. Addiction.

[12] Hayatbakhsh M.R., Flenady V.J., Gibbons K.S., Kingsbury A.M., Hurrion E., Mamun A.A., Najman J.M. (2011). Birth outcomes associated with cannabis use before and during pregnancy. Pediatr. Res..

[13] Van Gelder M.M.H.J., Reefhuis J., Caton A.R., Werler M.M., Druschel C.M., Roeleveld N. (2010). Characteristics of pregnant illicit drug users and associations between cannabis use and perinatal outcome in a population-based study. Drug Alcohol Dependence.

[14] Huizink A.C. (2014). Prenatal cannabis exposure and infant outcomes: Overview of studies. Prog. Neuro-Psych. Biol. Psych..

[15] Irner T.B., Teasdale T.W., Nielsen T., Vedal S., Olofsson M. (2012). Substance use during pregnancy and postnatal outcomes. J. Addict. Dis..

[16] Apter G., Devouche E., Gratier M. (2011). Perinatal mental health. J. Nerv. Ment. Dis..

[17] Brener N.D., Billy J.O.G., Grady W.R. (2003). Assessment of factors affecting the validity of self-reported health-risk behaviour amond adolescents: Evidence from the scientific literature. J. Adolescent Health.

[18] Brown J., Kranzler H.R., del Boca F.K. (1992). Self-reports by alcohol and drug abuse inpatients: Factors affecting reliability and validity. Brit. J. Addict..

[19] National Centre for Classification in Health (1999). The International Statistical Classification of Diseases and Related Health Problems.

[20] Centre for Health Record Linkage. http://www.cherel.org.au.

[21] Adhikari P. (2006). Socio-economic Indexes for Areas: Introduction, Use and Future Directions.

[22] Orr S.T., Reiter J.P., James S.A., Orr C.A. (2012). Maternal health prior to pregnancy and preterm birth among urban, low income black women in Baltimore: The Baltimore preterm birth study. Ethn. Dis..

[23] Helmbrecht G.D., Thiagarajah S. (2008). Management of addiction disorders in pregnancy. J. Addict. Med..

[24] Shor S., Nulman I., Kulaga V., Koren G. (2010). Heavy in utero ethanol exposure is associated with the use of other drugs of abuse in a high-risk population. Alcohol.

[25] Harrison P., Sidebottom A. (2009). Alcohol and drug use before and during pregnancy: An examination of use patterns and predictors of cessation. Matern. Child Health J..

[26] Birnbach D., Browne I.M., Kim A., Stein D.J., Thys D.M. (2001). Identification of polysubstance abuse in the parturient. Brit. J. Anaesth..

[27] Martini J., Knappe S., Beesdo-Baum K., Lieb R., Wittchen H-U. (2010). Anxiety disorders before birth and self-perceived distress during pregnancy: Associations with maternal depression and obstetric, neonatal and early childhood outcomes. Early Hum. Dev..

